# Impact of COVID-19 on Acute Appendicitis: Real-World Study at a Third-Level Hospital in Mexico City

**DOI:** 10.7759/cureus.90582

**Published:** 2025-08-20

**Authors:** Edgar A González-Macedo, Catalina Ortiz Monasterio, Domingo J Coutinho Thomas, Armando Torres Gómez, Martín Vega-de Jesús, Jorge G Obregón Méndez, César Decanini-Terán

**Affiliations:** 1 Surgery, American British Cowdray Medical Center, Mexico City, MEX; 2 Colorectal Surgery, American British Cowdray Medical Center, Mexico City, MEX

**Keywords:** acute appendicitis, appendicitis, complications, covid-19, pandemic, real-world, sars-cov-2

## Abstract

Background

Acute appendicitis (AA) is a common surgical emergency, with delayed diagnosis increasing the risk of complications. The COVID-19 pandemic disrupted healthcare access, potentially leading to more severe presentations of AA. This study assesses the pandemic’s impact on the severity and outcomes of AA.

Methods

This retrospective cohort study analyzed patients diagnosed with AA at the American British Cowdray Medical Center from November 2019 to July 2020. The patients were categorized into pre-pandemic (before March 23, 2020) and post-pandemic (after March 24, 2020) groups. Demographics, laboratory findings, intraoperative classification with American Association for the Surgery of Trauma (AAST) grades I-V, histopathology, and duration of hospital stay were compared.

Results

Of the 192 patients included in the study, 119 were in the pre-pandemic group and 73 in the post-pandemic group. Although the post-pandemic group had longer symptom duration before hospital admission, the difference was not statistically significant (p=0.1078). However, the post-pandemic group had significantly more cases of complicated appendicitis (p=0.0012) and perforated appendicitis (p=0.0171). Histopathological analysis showed that post-pandemic patients were 1.8 times more likely to have complicated appendicitis (OR=1.8, 95% CI:1.3-2.6). The duration of hospital stay remained similar between groups (p=0.2957).

Conclusions

The COVID-19 pandemic led to delayed AA presentation and increased disease severity. Public health measures should stress the importance of prompt medical evaluation to prevent complications. Further studies are needed to explore additional factors influencing delays in surgical emergencies.

## Introduction

Acute appendicitis (AA) is one of the most common indications for emergency surgical intervention and the leading cause of emergency abdominal surgical admissions. AA is a progressive pathological process, whose main pathways are infection and inflammation of the appendix. If left untreated, it can potentially advance to necrosis and perforation [1]. Delayed medical intervention increases the risk of severe complications, ranging from transmural necrosis to generalized peritonitis [2].

The COVID-19 pandemic significantly disrupted healthcare access and delivery, resulting in a decrease in medical consultations due to patient hesitancy and institutional infection control measures. Consequently, patients experiencing abdominal pain may have deferred seeking timely medical evaluation, potentially resulting in delayed diagnosis and management of AA. Additionally, the healthcare system overload during the pandemic may have further contributed to treatment delays [3-5].

This real-world study aims to evaluate the impact of the COVID-19 pandemic on the clinical presentation of AA, with a focus on potential delays in diagnosis and treatment at a private institution in Mexico City.

## Materials and methods

A retrospective cohort of patients diagnosed with AA and treated at the American British Cowdray Medical Center between November 1, 2019, and July 16, 2020, was analyzed. The study included all patients aged 16 years or older with a diagnosis of AA, while patients who underwent incidental appendectomy during another surgical procedure were excluded.

The patients were divided into two study groups: those diagnosed with AA before the declaration of the phase two contingency measures in Mexico City (November 1, 2019 to March 23, 2020), and those diagnosed after the declaration of the phase two contingency measures (March 24, 2020 to July 16, 2020).

The analysis included patient age, sex, duration of symptoms before admission to the emergency department, white blood cell (WBC) count, absolute neutrophil count, band count, and ultrasensitive C-reactive protein (CRP) levels at admission. Additionally, intraoperative findings, histopathological results, and length of hospital stay were evaluated. The intraoperative findings were classified into five grades, according to the American Association for Surgery of Trauma (AAST) classification [6] as follows: (1) Grade I: acute inflammation of the appendix; (2) Grade II: gangrenous appendix; (3) Grade III: perforated appendix with localized contamination; (4) Grade IV: perforated appendix with phlegmon or peri-appendicular abscess; and (5) Grade V: perforated appendix with generalized peritonitis.

Histopathological findings were categorized as fibrinopurulent appendicitis, gangrenous appendicitis, or perforated appendicitis. This study protocol was approved by the Research, Research Ethics, and Biosafety Committees of the American British Cowdray Medical Center, Mexico City (approval no. ABC-20-24).

Statistical analysis

Continuous variables were assessed for normality using the Shapiro-Wilk test. Variables with a normal distribution were reported as mean (standard deviation), while non-normally distributed variables are presented as median (interquartile range, minimum-maximum). Categorical variables are expressed as absolute and relative frequencies (percentages).

Comparisons between normally distributed variables were performed using the independent samples t-test, whereas non-normally distributed variables were analyzed using the Wilcoxon rank-sum test. Categorical variables were compared using Fisher’s exact or chi-square tests, depending on the expected cell counts. The strength of association between categorical variables was evaluated using odds ratios with 95% confidence intervals. A p-value ≤ 0.05 was considered statistically significant.

Statistical analyses were conducted using R software (version 4.0.2, R Foundation for Statistical Computing, Vienna, Austria, https://www.R-project.org/). The analyses were performed on the RStudio platform (version 1.3.959, Posit Software, Boston, MA) with the ggplot2 package (Wickham H (2016). ggplot2: Elegant Graphics for Data Analysis. Springer-Verlag New York. ISBN 978-3-319-24277-4, https://ggplot2.tidyverse.org).

## Results

A total of 218 patients were diagnosed with AA. After excluding 25 patients under 16 years old and one patient with incomplete clinical records, 192 patients were included in the analysis. Of these, 119 were treated before the implementation of the phase two pandemic measures (pre-group), and 73 were treated afterward (post-group). Both groups had similar demographic characteristics, as shown in Table 1.

**Table 1:** Demographic characteristics Values are presented as median (interquartile range or IQR, min-max) and absolute frequencies (%).

Characteristic	Pre-group (n=119)	Post-group (n=73)	p-value	Statistical value	Statistical analysis
Age	36 (24, 15-79)	34 (19, 16-77)	0.692	W = 4492	Wilcoxon rank-sum test
Female patients	58 (48.7%)	32 (43.8%)	0.5086	χ² = 0.43693 (1 g.l.)	Chi-square test
Male patients	61 (51.3%)	41 (56.2%)	0.5086		Chi-square test
COVID-19	0 (100%)	1 (1.4%)	0.1433	OR = ∞	Fisher’s exact test

Patients in the post-group had a longer time between symptom onset and diagnosis, but the difference was not statistically significant (p=0.1078). Ultrasensitive CRP levels were also higher in the post-group, though this was not statistically significant (p=0.5371). There were no significant differences between the groups in terms of WBC count, neutrophils, band cells, or duration of hospital stay (Table 2).

**Table 2:** Perioperative clinical characteristics and differences between the groups Values are presented as median (Interquartile range or IQR, min-max) and mean (SD). ^†^n=67 for pre-group; n=45 for post-group.

Variable	Pre-group (n=119)	Post-group (n=73)	Difference	p-value	Statistical value	Statistical analysis
Duration of hospital stay (h)	19 (24, 1-168)	24 (36, 3-240)	5	0.1078	W = 3744.5	Wilcoxon rank-sum test
Leukocytes (mil/µL)	12.49 (3.97)	12.81 (4.37)	0.32	0.5976	t = -0.52875 (df=190)	t-test for independent samples
Neutrophils (%)	9.72 (3.67)	10.16 (4.19)	0.44	0.4392	t = -0.77517 df=189	t-test for independent samples
Bands (%)	0 (2, 0-15)	1 (2, 0-14)	1	0.2330	W = 3935.5	Wilcoxon rank-sum test
CRP (mg/dL)^†^	1.42 (3.55, 0.01-34.48)	2.43 (6.10, 0.02-36.07)	1.01	0.5371	W = 1403	Wilcoxon rank-sum test

When analyzing surgical findings, more patients in the post-group had perforated appendicitis compared to the pre-group, and this difference was statistically significant (p=0.0171). When we combined cases of gangrenous and perforated appendicitis (both considered complicated appendicitis), the post-group had significantly more cases than the pre-group (p=0.0012), as shown in Tables 3, 4.

**Table 3:** Intraoperative findings in all the cases

Appendix type	Pre-group (n=119)	Post-group (n=73)	Difference	p-value	Statistical value	Statistical analysis
Inflamed	97 (81.5%)	44 (60.3%)	-53	0.02229	X-squared=11.413, df=4	Chi-square test
Gangrenous	8 (6.7%)	7 (9.6%)	-1			
Local contained perforation	2 (1.7%)	3 (4.1%)	1			
Phlegmon/abscess perforation	8 (6.7%)	13 (17.8%)	5			
Peritonitis perforation	4 (3.4%)	6 (8.2%)	2			

**Table 4:** Intraoperative findings with grouping of the cases with a gangrenous and perforated appendix *statistically significant; RM: Repeated measures; CI: confidence interval.

Appendix type	Pre-group (n=119)	Post-group (n=73)	Difference	RM (95% CI)	p-value*	Statistical value	Statistical test
Inflamed	97 (81.5%)	44 (60.3%)	-53	1.8 (1.3 – 2.6)	0.0012	X-squared=10.46, df=1	Chi-square test
Gangrenous + Perforated	22 (18.5%)	29 (39.7%)	7				

Histopathology results also showed a higher prevalence of cases with perforated appendicitis in the post-group, but this difference was not statistically significant (p=0.600). However, when we grouped both gangrenous and perforated appendicitis, patients in the post-group were 1.8 times more likely to have complicated appendicitis than those in the pre-group (OR=1.8, 95% CI:1.3-2.6), as shown in Tables 5, 6.

**Table 5:** Pathology results

Appendix type	Pre-group (n=119)	Post-group (n=73)	Difference	p-value	Statistical value	Statistical analysis
Fibrinopurulent	103 (86.6%)	53 (72.6%)	-50	0.04126	X-squared=6.3757, df=2	Chi-square test
Gangrenous	4 (3.4%)	3 (4.1%)	-1			
Perforated	12 (10.1%)	17 (23.3%)	5			

**Table 6:** Pathology results with grouping of cases with a gangrenous and perforated appendix RM: repeated measures; CI: confidence interval.

Appendix type	Pre-group (n=119)	Post-group (n=73)	Difference	RM (95% CI)	p-value	Statistical value	Statistical analysis
Fibrinopurulent	103 (86.6%)	53 (72.6%)	-50	2.43 (1.16-5.07)	0.0162	X-squared=5.7811, df=1	Chi-square test
Gangrenous + Perforated	16 (13.4%)	20 (27.4%)	4				

The duration of hospital stay was similar between the two groups (p=0.2957). Although the increased rate of perforated appendicitis in the post-group was not statistically significant, the data suggest a trend in that direction. A larger sample size may have helped clarify this association. This sample size was because the American British Cowdray Medical Center is a private institution and incidental appendectomy during another surgical procedure was excluded from this study.

## Discussion

AA remains one of the most common causes of acute abdomen and a leading indication for emergency surgery worldwide, even in the COVID-19 pandemic, with the potential for localized abscess formation or generalized peritonitis if left untreated [7,8].

Our findings indicate a trend toward delayed presentation among patients with AA during the COVID-19 pandemic. Although the time from symptom onset to hospital admission was longer in the post-pandemic group, this difference did not reach statistical significance. However, a significant increase in the incidence of complicated appendicitis, particularly perforated cases, was observed in this cohort. These findings suggest that even a modest delay in seeking medical care may contribute to disease progression, increasing the likelihood of perforation and subsequent complications.

The progression of appendicitis is known to be time-dependent, with previous studies reporting perforation rates of approximately 20% within the first 24 hours and up to 65% after 48 hours of symptom onset [7-9]. While our study did not assess the precise duration of symptoms before perforation, the higher incidence of complicated appendicitis in the post-pandemic group aligns with existing evidence that delays in presentation may significantly impact disease severity [10].

The COVID-19 pandemic introduced multiple barriers to healthcare access, including patient hesitancy to seek medical attention due to concerns about viral exposure and system-wide constraints related to hospital resource allocation in Mexico [11]. Our findings are consistent with reports from other international studies suggesting an increase in delayed diagnoses and a corresponding rise in the incidence of complicated appendicitis during this period [12-15].

Recent studies have explored additional dimensions of how the COVID-19 pandemic may have influenced the presentation and outcomes of AA. A pediatric cohort study suggested a potential link between prior SARS-CoV-2 infection and the subsequent development of AA, with a higher proportion of cases presenting within three months of infection and a non-significant trend toward increased rates of complicated appendicitis among unvaccinated children. While causality remains unproven, these findings raise important questions about post-viral inflammatory responses that may influence appendiceal pathology [16]. In contrast, a retrospective study from Lebanon found no significant differences in the severity, diagnostic imaging, or surgical outcomes of appendicitis cases between pre-pandemic and pandemic periods, indicating that healthcare access and patient behavior may have remained stable in certain populations [17]. Similarly, a US-based study from a major COVID-19 epicenter observed a marked decline in appendicitis presentations during peak-pandemic months, but reported no significant change in complication rates or surgical management [18]. These varying international experiences highlight the multifactorial nature of healthcare access, patient behavior, and disease presentation during the pandemic, and underscore the importance of contextual factors in interpreting the impact of global health crises on emergency surgical conditions such as appendicitis.

## Conclusions

The findings of this study suggest that the COVID-19 pandemic contributed to delayed presentations and a corresponding increase in the incidence of complicated appendicitis, particularly perforated cases. Although the time from symptom onset to hospital admission was longer in the post-pandemic group, the most significant clinical consequence was a higher rate of disease severity at the time of presentation. These results highlight the critical importance of timely medical evaluation for suspected appendicitis to mitigate the risk of progression to more severe forms and associated morbidity.

In light of the time-sensitive nature of AA, public health strategies must prioritize clear communication about the importance of not delaying emergency care, even amid global crises. Furthermore, emerging research suggests that a previous SARS-CoV-2 infection may also play a role in the presentation and complexity of appendicitis, particularly in pediatric populations, though further investigation is needed. Future multicenter studies with larger and more diverse populations are essential to confirm these trends and to acquire a better understanding of the interplay between infectious disease dynamics, healthcare access, and surgical emergencies.
